# Metabolism Is Central to Tolerogenic Dendritic Cell Function

**DOI:** 10.1155/2016/2636701

**Published:** 2016-02-11

**Authors:** Wen Jing Sim, Patricia Jennifer Ahl, John Edward Connolly

**Affiliations:** ^1^Institute of Molecular and Cell Biology, Agency for Science, Technology and Research, Singapore 138673; ^2^Institute of Biomedical Studies, Baylor University, Waco, TX 76712, USA

## Abstract

Immunological tolerance is a fundamental tenant of immune homeostasis and overall health. Self-tolerance is a critical component of the immune system that allows for the recognition of self, resulting in hyporeactivity instead of immunogenicity. Dendritic cells are central to the establishment of dominant immune tolerance through the secretion of immunosuppressive cytokines and regulatory polarization of T cells. Cellular metabolism holds the key to determining DC immunogenic or tolerogenic cell fate. Recent studies have demonstrated that dendritic cell maturation leads to a shift toward a glycolytic metabolic state and preferred use of glucose as a carbon source. In contrast, tolerogenic dendritic cells favor oxidative phosphorylation and fatty acid oxidation. This dichotomous metabolic reprogramming of dendritic cells drives differential cellular function and plays a role in pathologies, such as autoimmune disease. Pharmacological alterations in metabolism have promising therapeutic potential.

## 1. Introduction

Immune homeostasis is achieved when there is a balance between immunogenicity to nonself or pathogens and tolerance to self. Amongst many lymphocytes involved, dendritic cells (DCs) play an important role in both the innate and adaptive immune response. DCs originate from hematopoietic progenitor cells (HPCs) and contribute to immunity by recognition of pathogenic signals. Upon activation by Toll-like receptor (TLR) binding, DCs migrate from the periphery into lymph nodes during a maturation process. DCs can act as antigen-presenting cells (APCs) by efficiently presenting peptide-major histocompatibility complex (MHC), molecules to antigen-specific T cells which then eliminate pathogens [1]. Protection against pathogenic invasion is important, but it is also critical for immune system to be at the very least nonresponsive to self, a concept known as tolerance. Central tolerance is a deletional process where high affinity reactive T cells are eliminated [2]. Peripheral tolerance is the combination of inducing anergy in self-reactive T cells that escape the thymus and the suppressive action of regulatory T cells [3]. Specific types of DCs, the tolerogenic dendritic cells (tol-DCs), are critical in maintaining tolerance. Defects in self-tolerance play a role in autoimmune diseases and autoinflammatory diseases.

In recent years, cellular metabolism has been identified as a key component in immune cell function. Decades of research have led to the characterization of cellular metabolism as a vast network of biochemical processes important for energy production and cell fate determination [4]. Revolutionary advances in mass spectrometry, high performance liquid chromatography (HPLC), and extracellular flux analysis have opened up the field of immune bioenergetic analysis [5]. Studies have revealed fundamental metabolic differences within human peripheral blood leukocytes and their component subsets [6]. Furthermore, functional activity of these immune cells can be altered with changes in metabolic reprogramming. This review will focus on tol-DCs, metabolic reprogramming by pharmacological agents, and their potential use in the clinic.

## 2. Immunologic Tolerance

The function of immune system is to defend an organism from pathogenic invasion. Immunologic tolerance refers to an ability to suppress self-reactivity and control the response to prolonged and persistent infection. Tolerance is an active process involving multiple cellular subsets to constantly control self-reactivity. During an ongoing immune response, mechanisms are required to tightly regulate self-reactivity in a spatial and time dependent manner to reduce collateral tissue damage. Breakdown in tolerance results in serious pathology like autoimmune diseases, allergies, and graft rejections. In mammals, tolerance checkpoints occur mechanistically at two levels: centrally and peripherally.

Central tolerance acts as a first line of defence against autoimmunity. The chief mechanism of central tolerance is the deletion of autoreactive T cells in the thymus. This process is aided by thymic DCs and thymic medullary epithelial cells which present self-peptide-MHC complexes to T cells. T cells first undergo positive selection followed by negative selection during T cell development. Under positive selection, T cells with low T cell receptor (TCR) expression or an inability to react with MHC molecules are removed. Any self-reactive T cells are deleted from the T cell repertoire under negative selection when they react strongly with self-peptide-MHC complexes presented on thymic DCs [7, 8]. Despite an effective mechanism of limiting self-reactivity, T cells with moderate or low affinity may survive central tolerance scrutiny and enter the periphery.

Secondary peripheral mechanisms are required to suppress the activation of any remaining autoreactive cells. DCs are crucial in maintaining tolerance in the periphery. Constitutive ablation of all DCs in mice resulted in the development of spontaneous fatal autoimmunity under steady state conditions [9]. DCs are vital to the induction of T cell anergy in which T cells become functionally inactivated following an antigen encounter. In 2002, Bonifaz et al. showed that antigen delivery by anti-DEC-205 antibodies to DC induced CD8^+^ T cell tolerance [10]. T cell activation requires T cells to first recognize the appropriate MHC-peptide complex followed by costimulatory signals from DCs to proliferate and differentiate. In the absence of costimulatory signals, self-reactive T cells do not proliferate when they encounter self-peptide-MHC complexes and remain unresponsive towards primary and secondary stimulation, inducing T cell anergy. Self-reactive T cell activation may also be suppressed by regulatory T cells (Tregs). Natural Tregs are thymically derived and self-antigen-specific. Upon recognition of their specific antigen, they can broadly suppress nearby effector T cells in a nonspecific manner. Researchers also demonstrated that anti-DEC-205 antibodies targeted to immature DC or peptide-loaded mature DCs are potent inducers of Tregs yielding T cell tolerance, indicating that these cells may be the actual in vivo effectors [3, 11]. Peripheral tolerance is a dynamic multicellular process and plays a key role in keeping autoreactivity under surveillance.

## 3. Tolerogenic DCs

DCs form a critical link between the innate and adaptive immune systems and their state of maturation determines an immunological or tolerogenic outcome. In the presence of specific signals, immature DCs (iDCs) are differentiated to Th1/2/17/9 or TfH inducing state, while, in response to tolerogenic signals, iDCs are matured toward an alternate, Treg-inducing state [12]. Once generated, these cells integrate peripheral tolerogenic signals and inhibit T cell autoreactivity, thereby promoting peripheral tolerance and maintenance of immune homeostasis.

Tol-DCs can be characterized by their surface markers and cytokine profile. Generally, tol-DCs express lower levels of surface MHC class II and costimulatory molecules relative to immunogenic DC, a state often referred to as “semimature” [21–24]. These cells can be further characterized by the specific expression of surface immunoreceptor tyrosine-based inhibitory motifs (ITIMs) containing receptors such as Fc gamma receptor IIb (CD32b), Ig-like inhibitory receptors (ILT3/ILT4), and paired immunoglobulin-like receptor (PILR) [25]. They also express immunomodulatory molecules like PD-L1 (ligand of programmed death 1) [26], heme oxygenase 1, human leukocyte antigen G (HLA-G) [27, 28], CD95L, galectin-1 [29], and DC-SIGN (Dendritic Cell-Specific Intercellular adhesion molecule-3-Grabbing Nonintegrin) [12, 30, 31]. Interestingly, a number of genes involved in metabolism seem to play a critical role in tol-DC biology such as indoleamine 2,3-dioxygenase (IDO), IL-27 [32], and nitric oxide (NO) [12, 31]. The production of immunosuppressive factors including IL-10 and transforming growth factor-beta (TGF-b) can contribute to their function by promoting the expansion of Tregs and directly inhibiting T cell response. It is important to note that this phenotype is largely resistant to subsequent activation signals indicating it is not a transient differentiation state.

IL-10 is believed to play a pivotal role in regulating the expression of both immune-inhibitory receptors and cytokines during tolerogenic DC maturation process. A number of studies indicate that IL-10 regulates the expression of ILTs in DCs and monocytes [33, 34]. Treatment of myeloid DCs with exogenous IL-10 displays high levels of ILT3 and ILT4 surface expression [25]. Transduction of ILT3 and ILT4 in DCs results in generation of Foxp3^+^ Tregs and inhibition of alloproliferation [35]. IL-10 signaling also induces the expression of ILTs orthologue paired PILR-alpha [33, 36]. PILRs block the access of CD8 molecules to MHC-I and it has been demonstrated that PIR-B-deficient DCs result in Cytotoxic T Lymphocytes (CTL) activation and accelerated graft and tumor rejection [37]. IL-10 is crucial in regulating PD-L1 expression on tol-DCs. Ligation of PD-1/PD-L1 leads to the recruitment of SHP phosphatase which plays an important role in inhibiting T cell response [38, 39]. Exogenous addition of IL-10 in DC culture from normal donor upregulated PD-L1 surface expression, while IL-10 blockade with neutralizing antibody reversed upregulated PD-L1 expression in DCs from tumor patients [40]. Blocking PD-L1/PD-1 signaling pathway augmented HIV-specific CD4 and CD8 T cell function in chronic HIV infection [41]. IL-10 has also been shown to upregulate the expression of CD95L [42]. CD95/CD95L mediated apoptosis in T cells is important for the maintenance of peripheral tolerance and termination of an ongoing immune response [43]. IL-10 has also been shown to regulate the expression of IDO, an enzyme of tryptophan catabolism, in tol-DCs. The presence of IL-10 during DC maturation prevented IFN-*γ*-induced downregulation of IDO, resulting in sustained expression of functional IDO even in mature, IFN-*γ*-activated DCs [44]. Transduction of IDO gene into DCs suppressed allogeneic T cell proliferation in vitro [45]. IL-10 also regulates the expression of other tolerogenic DC markers, such as HLA-G, Inhibin Beta A (INHBA), Aquaporin 9 (AQP9), and Signaling Lymphocytic Activation Molecule (SLAM) [33], which are believed to be expressed on many tolerogenic DCs. Their functions as related to tolerance induction are unclear at this point.

There are differing opinions on the role of inhibitory cytokine TGF-b in the induction of DC tolerogenic state. An elegant study by Flavell Laboratory using a dominant negative form of TGF-beta receptor II under the control of CD11c promoter (CD11c^−^ dnTGFbetaRII) showed that DC homeostasis is independent of TGF-beta signaling [46]. However, subsequent studies show that TGF-b signaling does play a role in DC tolerogenesis. TGF-b1 gene modified bone marrow-derived immature DCs display decreased IL-12 secretion and alloantigen-specific T cell unresponsiveness in vitro and in vivo [47]. Pallotta et al. demonstrated that exogenous addition of TGF-b induces the expression of IDO in DCs, supporting spontaneous tolerogenesis [48, 49]. It has also been shown that TGF-b treatment increased the expression of PD-L1 in DCs, resulting in T cell apoptosis and Treg expansion [50]. Signal transducers mediated the increment in PD-L1 and activators of transcription 3 (STAT3) and blockade of STAT3 significantly decrease PD-L1 expression. TGF-b pulsed bone marrow-derived DCs result in a decrease in IL-12p70 production and inhibited allogenic T cells, leading to long-term survival of the graft [51].

Tol-DCs can also be generated ex vivo using several pharmacological agents such as dexamethasone (DEX), rapamycin, vitamin D_3_, or Vit D_3_/Dex combination [52–54]. These tolerogenic DCs are semimature in phenotype and possess the ability to suppress alloreactive response. DEX is an immunosuppressive glucocorticoid. DEX-polarized tolerogenic DCs are able to inhibit T cell proliferation and cytokine production as well as promote functional Treg expansion [55]. DEX treated DCs decrease expression of costimulatory molecules and increase secretion of IL-10 while decreasing IL-12 [56, 57]. Rapamycin is an inhibitor of the Ser/Thr protein kinase, mammalian target of rapamycin (mTOR), and widely used as an anticancer drug and an immunosuppressant. Rapamycin-treated DCs display a reduction in CD40, CD80, and CD86 expression and T cell hyporesponsiveness [58]. Rapamycin pulsed DCs promote the expansion of functional Treg [59]. Vitamin D_3_ is a pleiotropic hormone, which regulates calcium homeostasis, promoting innate immunity while inhibiting adaptive immunity. Exogenous treatment with Vit D_3_ results in high expression levels of PD-L1 in DCs, suppressing T cell proliferation [60]. Vit D_3_ treated DCs induce ILT3 expression and result in ILT3 dependent CD4^+^Foxp3^+^ regulatory T cells expansion [61] and alloreactive T cell inhibition [62]. Ligation of Vit D_3_ to vitamin D receptor (VDR) significantly increases NF-*κ*B promoter binding affinity and inhibits NF-*κ*B expression and activation, promoting tolerogenic DCs [63]. NF-*κ*B inhibitor, BAY 11-7082, and treated DCs display low expression of MHC class II and CD40 molecules and in vivo injection of BAY 11-7082-treated DC induces IL-10 producing CD4^+^ regulatory T cells [64]. Protein kinase C inhibitors such as bisindolylmaleimide I, GO 6983, and RO 32-0432 inhibit NF-*κ*B activation in DCs, giving rise to tolerogenic DCs [65]. *β*-catenin activation drives tolerogenic DCs cell fate and it has been shown that disruption of E-cadherin signaling leads to the activation of *β*-catenin, giving rise to tolerogenic DCs [66]. DC-specific deletion of *β*-catenin increased proinflammatory cytokine production [67]. Interestingly, all these pathways target different steps in DC differentiation and activation and yet they converge to generate cells with functionally similar characteristics. It is important to determine the underlying biological process driving this common differentiation.

### 3.1. Metabolism

The role of metabolism in underpinning immune cell function has become an area of active research over recent years. In living organisms, cellular metabolism is critical for the production of energy in the form of ATP, as well as cellular maintenance and proliferation [4]. Glycolysis is a metabolic process that breaks down glucose to rapidly produce ATP. Intermediates of glycolysis can enter the Pentose Phosphate Pathway (PPP), which generates reductive capability and anabolic building blocks. Oxidative products of this pathway can feed back into glycolysis and affect cellular function [68, 69]. In most cells, oxidative phosphorylation (OXPHOS) produces the bulk amount of ATP molecules [70]. Otto Warburg has described the shift away from OXPHOS and towards aerobic glycolysis despite the presence of oxygen as the Warburg effect [71]. The shift in cell metabolism can be explained by the requirement for quick biosynthesis in contrast to efficient energy production. A variety of immune cells have been shown to display different metabolic priorities. With recent advances in metabolic analysis including the extracellular flux analyzers, cellular oxygen consumption rate (OCR) and extracellular acidification rate (ECAR) can be determined by generating bioenergetic profiles of mitochondrial respiration and glycolysis, respectively [6]. The analysis of peripheral blood from healthy donors shows distinct metabolic profiles in monocytes, lymphocytes, and platelets determined by parameters including basal respiration, ATP-linked respiration, proton leak, reserve capacity, and non-mitochondrial respiration. In these studies, differences in the metabolic priorities of immune cells become apparent. Neutrophils seemingly dedicate most energy production to glycolytic metabolism, lymphocytes mainly use oxidative phosphorylation, and monocytes show a degree of both glycolytic and oxidative pathways [72]. Studies looking at differential metabolic programs in lymphocytes demonstrate the importance of metabolism for immune function. In T cells, metabolism defines the immune response, where memory T cells have been revealed to rely on increased FAO and glucose [73]. The involvement of mitochondria during T helper cell (T_H_) activation has also become evident. During Ca^2+^-dependent activation of T_H_, an immunological synapse is formed between T_H_ and APCs. This is associated with a relocalization within the T_H_ cell structure, bringing mitochondria closer and increasing the influx of Ca^2+^, indicating mitochondrial involvement in T_H_ activation [74]. Additionally, Chang et al. have shown the role of T cell metabolism in tumor growth, where a decrease in glycolysis and nutrient availability for T cells fuels tumor progression [75]. The cellular metabolic state dictates downstream function for many immune cells.

### 3.2. Metabolism in DC

Changes in cellular metabolism are important in many aspects of DC development and function. During DC maturation, the metabolic profile of precursors and differentiating DCs is different, shifting from glycolysis to OXPHOS [76]. Studies show that this involves reactive oxygen species, as well as an increase in expression of mitochondrial respiratory enzymes, ATP content, and antioxidant capacity [77]. Similarly, resting and activated DCs show differences in metabolic priorities. Maturation of DCs by a variety of TLR stimuli initiates specific signaling cascades, which feed into metabolic regulation. The AKT pathway activates Hexokinase 2 to boost glycolysis and the TCA cycle, resulting in increased PPP activity and the formation of citrate for fatty acid synthesis. The NF-*κ*B signaling pathway is also triggered and can cause stress in the endoplasmic reticulum, which stimulates the constitutively active unfolded protein response (UPR) and promotes fatty acid synthesis [76]. Recently, Cubillos-Ruiz et al. revealed that, in tumor-associated DCs, downstream activation of X-box-binding protein 1 (XBP1) by UPR and the related production of lipid bodies impede DC antigen-presenting function and effectiveness in initiating T cell responses [78]. With this shift towards glycolysis in activated DCs, glycolytic intermediates can promote PPP which support biosynthesis of nucleotides for increased protein output and the generation of NADPH. Glycolytic intermediates can also enter the TCA cycle and support lipid membrane production and macromolecule biosynthesis [79]. Furthermore, during LPS activation, OXPHOS and FAO are decreased due to the upregulation of inducible nitric oxide synthase (iNOS) and inhibition of 5′ adenosine monophosphate-activated protein kinase (AMPK), respectively [71]. Everts et al. show that iNOS can block OXPHOS and NO can shut down mitochondrial function [80]. Therefore, the different metabolic profiles in DCs are a key factor in differential DC functions. This raises the possibility of altering the immune response by influencing DC metabolism for therapeutic purposes in diseases, such as autoimmunity and cancer.

**Figure 1:**
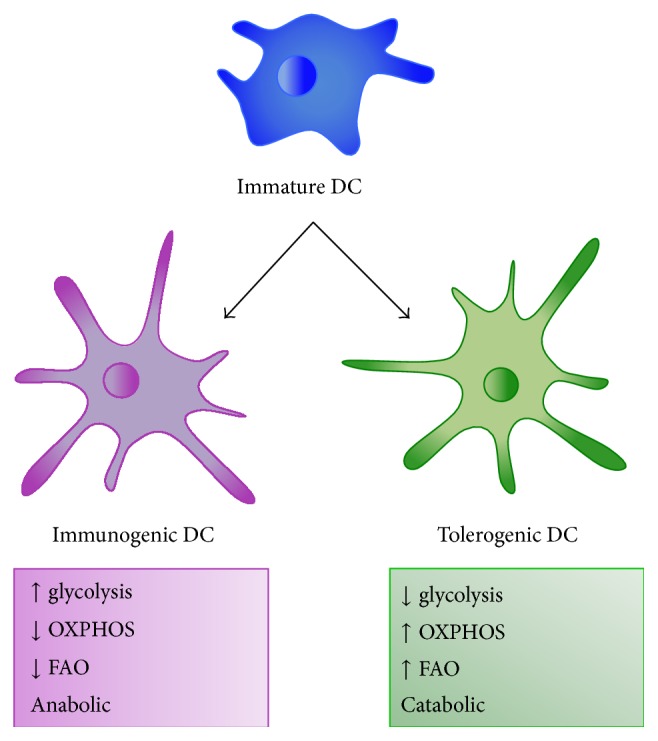
Differentiation of dendritic cells. Immature dendritic cells (DCs) can mature into either activated, immunogenic DCs that induce T_H_1/T_H_2/T_H_17 for T cell function and elimination of pathogens or tolerogenic DCs that induce the expansion of T regulatory cells and T cell unresponsiveness for immune tolerance. Immature DCs mature into immunogenic DC with a shift in metabolism towards glycolysis, which is associated with increasing biomass for effector function. Tolerogenic DC, on the other hand, shifts cellular metabolism towards OXPHOS (oxidative phosphorylation) and favors FAO (fatty acid oxidation). This catabolic and highly energetic profile may be related to energy required for active suppression and protein degradation.

Tol-DCs also show a distinct metabolic profile (Figure 1). In recent studies by Malinarich et al., the expression of metabolic pathway related genes shows fundamental differences within DCs, with a focus on tol-DCs. The studies demonstrate an increased expression of genes related to OXPHOS, especially of electron transport chain complexes II and IV. Additionally, IL-10, a characteristic cytokine of tol-DCs, blocks the shift in metabolism at TLR stimulation to glycolysis and favors OXPHOS [81]. Moreover, high mitochondrial activity and increased ROS production are seen in tol-DCs. This mitochondrial activity is associated with an increase in FAO and the utilization of triglycerides as a carbon source. Inhibition of the pathway leads to a loss of tolerogenic capacity. Inhibition with etomoxir increased responder T cell activation and caused a dramatic decrease in OCR [82]. Similar to resting T cells, the catabolic profile and high-energy demand in tol-DCs may be related to their active suppressive function. Tryptophan breakdown plays a role in peripheral tolerance [83] and the metabolic enzyme arginase-1 has been shown to control DC differentiation in mice [84]. Lying upstream of immune function, the manipulation of DC metabolism may be used for therapeutic purposes.

### 3.3. Pharmacological Manipulation of DC Metabolism for DC Vaccines

**Figure 2:**
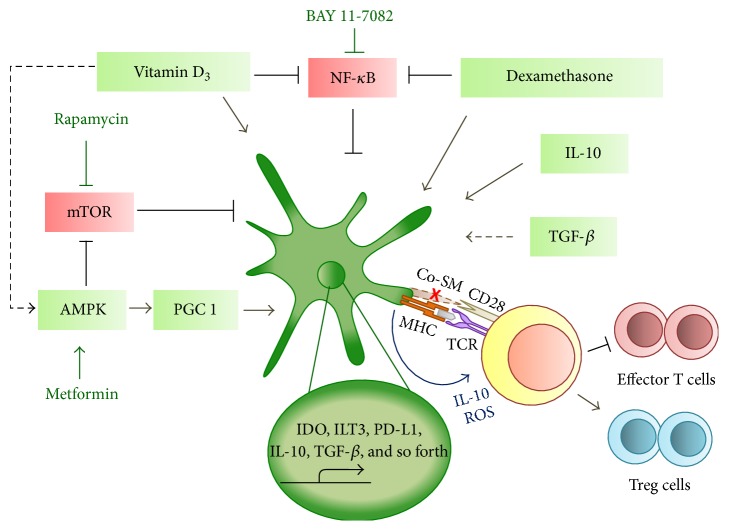
Induction of tolerogenic dendritic cells by cytokines and pharmacological agents. Tolerogenic dendritic cells can be induced by TGF-*β* (transforming growth factor-beta), IL-10 (interleukin-10), and pharmacological agents dexamethasone, BAY 11-7082, vitamin D_3_, rapamycin, and metformin which regulates dendritic cell metabolism. The induction of tolerogenic dendritic cells leads to an increase in IDO (indoleamine 2,3-dioxygenase), ILT3 (Ig-like inhibitory receptor 3), PD-L1 (ligand of programmed death 1), IL-10, and TGF-*β* transcription and expression. The downregulation of Co-SM (costimulatory molecules) on tolerogenic dendritic cells and the subsequent release of IL-10 and ROS (reactive oxygen species) result in the inhibition of T cells alloproliferation and the expansion of Treg (T regulatory cells), inducing tolerance.

Extensive research on long-term graft acceptance led to the identification of tolerogenic myeloid populations including DCs [85]. Many pharmacological agents such as Vit D_3_, DEX or Vit D_3_/DEX combination, or rapamycin can be used to generate tol-DCs (Figure 2). Vit D_3_ induces changes in proteins involved in iron metabolism, TCA cycle, OXPHOS, and the PPP. A recent paper demonstrated that Vit D_3_ acts synergistically with metformin to increase the activation of AMPK pathway [86]. Being a metabolic sensor, AMPK is able to detect fluctuations in the AMP : ATP ratio. Activation of AMPK inactivates acetyl-CoA carboxylase, releasing the inhibition on carnitine palmitoyltransferase-1 (CPT-1). This promotes catabolic metabolism, increasing FAO and OXPHOS [70, 87–89]. Pharmacological activation of AMPK signaling also promotes mitochondrial biogenesis, increasing mitochondrial OXPHOS by peroxisome proliferator-activated receptor gamma coactivator (PGC) 1-*α* [70, 87]. Resveratrol has been shown to enhance PGC-1*α* activity and is being used for the induction of tolerogenic DCs [70, 90]. Vit D_3_ generated tol-DCs are able to suppress TLR-driven activation and glucose consumption [91]. Tolerogenic DCs generated with VDR agonists have been used as DC vaccines in clinical treatment of psoriasis, a Th1 and Th17 cell-mediated autoimmune disease of the skin [13]. Yates et al. demonstrated that infusion of Vit D_3_ metabolically modified DCs into TCR-Tg RAG^−/−^ mice that accepted a skin transplant resulted in de novo generation of CD25^+^Foxp3^+^ Tregs and dominant transplant tolerance. Mice that were previously rendered tolerant not only retained their existing grafts but also accepted fresh grafts indefinitely [14].

Glucocorticoid receptor activation leads to a cascade of anti-inflammatory responses in a variety of immune cells. In DCs, DEX administration regulates the MHC-II antigen presentation pathway and the proteins involved in the response to stress [92, 93]. DEX is able to inhibit the expression of iNOS and NO production, often increased in inflammatory diseases [94]. DCs generated with DEX have been used to minimize allograft rejection. Emmer et al. demonstrated that preinjection of LPS-matured DEX-DCs resulted in prolonged survival of a completely MHC-mismatched heart allograft in the transplant recipient [15]. A combination of Vit D_3_ and DEX has also been used to generate tol-DCs. These Vit D_3_/DEX-DCs display a Vit D_3_ induced metabolic profile and stress response similar to DEX-DCs [52]. These DCs are less sensitive to death by nutrient starvation and have robust antioxidative machinery. It has been shown that Vit D_3_/DEX generated DCs are more potent in inhibiting allogenic T cell proliferation than Vit D_3_ or DEX-DCs [95]. Pedersen et al. demonstrated that infusion of Vit D_3_/DEX treated DCs into CD4^+^CD25^−^ T cell transfer SCID mice resulted in suppression of colitis [16].

mTOR is a key regulator of glycolysis and anabolic metabolism [96] and its inhibition with rapamycin has been shown to induce catabolic metabolism and the generation of tolerogenic DCs. A number of studies have highlighted the potential of metabolically modified rapamycin-treated DCs in the induction of transplant tolerance. Injection of alloantigen-pulsed RAPA-DC vaccine into mice before transplantation significantly prolonged a full MHC-mismatched organ allograft [17, 97]. More strikingly, when CD4^+^ T cells from RAPA-DC vaccinated heart-graft recipient mice were adoptively transferred to naïve mice, resistance to transplant rejection could also be transferred [18].

## 4. Conclusion and Future Perspectives

Induction of full tolerance remains an elusive goal in clinical organ transplantation and in the management of autoimmune diseases. Based on the promising efficacy in animal models, several groups have begun initiating clinical trials of tol-DC vaccination to treat autoimmune diseases. A phase I clinical trial has started using tol-DC vaccines to reduce islet-specific inflammation in type-1 diabetes [19, 98]. Several groups are starting DC vaccination clinical trials for treating Rheumatoid Arthritis (RA) using BAY 11-7082-treated DC loaded with citrullinated peptides derived from RA patients [20]. Another DC vaccine trial is underway using autologous DC metabolically modified with dexamethasone and vitamin D_3_ and loaded with synovial fluid [99]. Cell based tolerogenic DC vaccinations, including those mentioned above, may prove uniquely effective in restoring immune homeostasis in patients with autoimmune disease. To date, there is no published report on clinical trials using tolerogenic DC vaccines in transplantation, but given the great need for novel tolerogenic strategies in this field, studies may be forthcoming.

Most trials to date have used ex vivo modulated DCs for vaccination. Antigen-pulsed DCs treated with pharmacological agents might not reflect the complexity of natural tol-DCs in vivo. It should be noted that, in the clinic, this approach has shown efficacy and has the advantage that these cells can be generated in sufficient amounts for multiple injections. Loading with specific antigens may improve clinical outcome; however, this poses a challenge in diseases with many autoantigens, such as systemic lupus erythematosus. There has been a growing body of work focused on using antibodies to deliver tolerogenic signals directly to DCs in vivo, thereby bypassing the need for ex vivo manipulation and the potential side effects of immunosuppressive drugs [11, 100, 101]. It remains to be seen if the future of tolerogenic DC vaccination lies in this generation of complex biologic targeting molecules.

There is growing evidence that distinct metabolic reprogramming lies at the heart of DC differentiation, acting as a master regulatory switch in determining immunogenic or tolerogenic DC cell fate. Given the broad availability of pharmacologic compounds, which target this axis, metabolically modulated tol-DCs are promising tools for tolerogenic vaccination in the clinic. DC vaccination to prevent or to inhibit immune activation is highly sought after in allergy, autoimmunity, and transplantation medicine. Our understanding of the mechanism to which metabolism regulates cellular processes in DCs is increasing. Evidence from a multitude of animal models strongly demonstrated the efficiency of metabolically modulated tol-DC vaccines in allergic and autoimmune diseases as well as in transplantation (Table 1). However, for DC-based tolerogenic vaccination in man, only a few clinical trials have been performed for inflammatory and autoimmune disorders (Table 1). Future studies are required to identify the important metabolic targets which can be manipulated to induce tol-DCs for vaccination and facilitate their rapid translation to the clinic. Metabolic modification of DCs yielding phenotypically stable tol-DCs with migratory capacity, combining DC vaccines with autoantigens, and timely and accurate delivery of DC vaccines to target sites are all important approaches with potential to further enhance vaccine efficacy.

## Figures and Tables

**Table 1:** Dendritic cell therapeutics.

Condition	Organism	Adjuvant	Metabolic modulator	Immune response	References
Psoriasis	Human	1,25(OH)_2_D_3_	Yes	Increased CD4^+^CD25^+^ suppressor T cells	[13]

Skin transplant	Mouse	1,25(OH)_2_D_3_	Yes	Increased CD25^+^Foxp3^+^ Tregs	[14]

Allograft rejection	Mouse	Dexamethasone	Yes	Increased ratio IL-10/IL-12	[15]

Colitis	Mouse	1,25(OH)_2_D_3_ & dexamethasone	Yes	Lower CD4^+^CD25^−^ T cell response	[16]

Heart graft	Mouse	Rapamycin	Yes	Inhibiting T cell IL-2 & IFN-*γ* production	[17]

Heart graft	Mouse	Rapamycin	Yes	Stimulating CD4^+^CD25^+^Foxp3^+^ Treg	[18]

Type-1 diabetes	Human	Antisense oligonucleotides targeting CD40, CD80, and CD86	No	Stimulating B220^+^ CD11c^−^ B-cells	[19]

Rheumatoid Arthritis	Human	BAY 11-7082	No	Higher ratio Treg/effector T cells	[20]

## Abbreviations

AMPK: 5′ adenosine monophosphate-activated protein kinase
APC: Antigen-presenting cell
DCs: Dendritic cells
DC-SIGN: Dendritic Cell-Specific Intercellular adhesion molecule-3-Grabbing Nonintegrin
DEX: Dexamethasone
ECAR: Extracellular acidification rate
FAO: Fatty acid oxidation
HPCs: Hematopoietic progenitor cells
HLA-G: Histocompatibility antigen, class I, G
HPLC: High performance liquid chromatography
IL-10: Interleukin-10
IL-27: Interleukin-27
ILT3: Immunoglobulin-like transcript 3
ILT4: Immunoglobulin-like transcript 4
iDCs: Immature DCs
IDO: Indoleamine 2,3-dioxygenase
iNOS: Inducible nitric oxide synthase
MHC: Major histocompatibility complex
mTOR: Mammalian target of rapamycin
NO: Nitric oxide
OXPHOS: Oxidative phosphorylation
OCR: Oxygen consumption rate
PBMCs: Peripheral Blood Mononuclear Cells
PD-L1: Ligand of programmed death 1
PPP: Pentose Phosphate Pathway
Tregs: Regulatory T cells
TCR: T cell receptor
T_H_: T helper cell
TGF-b: Transforming growth factor-beta
TLR: Toll-like receptors
TNF: Tumor Necrosis Factor
Tol-DC: Tolerogenic DC
TCA: Tricarboxylic Acid
UPR: Unfolded protein response
VDR: Vitamin D (1,25-dihydroxyvitamin D_3_) receptor
Vit D_3_: Vitamin D (1,25-dihydroxyvitamin D_3_).
